# Communication in the face of death and dying - how does the encounter with death influence the patient management competence of medical students? An outcome-evaluation

**DOI:** 10.1186/s12909-021-03060-5

**Published:** 2022-01-10

**Authors:** T. Thyson, M. Schallenburger, A. Scherg, A. Leister, J. Schwartz, M. Neukirchen

**Affiliations:** 1 Interdisciplinary Centre of Palliative Care, University Hospital, Heinrich Heine University, Duesseldorf, Germany; 2 University Hospital Duesseldorf, Interdisciplinary Centre for Palliative Medicine, Moorenstr. 5, 40225 Duesseldorf, Germany; 3 Department of Anaesthesiology, University Hospital, Heinrich Heine University, Duesseldorf, Germany

**Keywords:** Communication at the end of life, Blended learning, Meeting with real-world patients, Palliative medicine

## Abstract

**Background information:**

As part of an elective course, the Interdisciplinary Centre for Palliative Medicine at Duesseldorf University Hospital offers medical students the opportunity to personally meet and talk to a seriously ill patient on one or more occasions. The future physicians are provided with an opportunity to broaden their professional competence, i.e. their knowledge and skills in patient-centred communication at the end of life, and enhance their personal competence, for example in how to professionally handle their own emotions. A topical e-learning module helps the students to prepare for the meetings, and writing a reflection paper forms the basis for the concluding reflection seminar.

**Objectives:**

The study’s objective is a global and outcome-based evaluation of the elective blended-learning course that provides real-world patient interaction. The outcome-based evaluation or outcome assessment aims to objectively evaluate changes identified in knowledge, skills and attitude among the participants of the elective-course. Furthermore, the evaluation aims to answer the question of whether changes especially in attitude (social skills and self-competence) should be expected after the students have met with severely ill or dying patients.

**Method:**

On two questionnaires specifically developed for this survey the students were able to provide a global rating of the elective course and describe their learning gains in palliative care. The students’ learning gains were measured by means of 14 items reflecting the specific educational objectives of the offered elective course. Using the German school grading system as a rating scale, the students assessed their learning progress by retrospectively evaluating their skills before and after completion of the elective course (Comparative Self-Assessment, CSA).

**Results:**

In the time from April 2018 till March 2020, 62 students participated in the evaluation. Overall, learning progress among students could be observed across all areas of competence, and in 50% of all retrospective self-assessment items the learning gains were ≥ 50%. The highest learning gain (63.6%) was observed in the students’ ability to meet a severely ill patient without fear. The lowest learning gain was observed when students had to confront and accept their own mortality.

**Conclusions:**

The offered elective course supports students in achieving social and self-competence development goals. According to the obtained results, contact with real-world patients helps mould the students’ attitude.

## Introduction

“Professional competence is the habitual and judicious use of communication, knowledge, technical skills, clinical reasoning, emotions, values, and reflections in daily practice for the benefit of the individual and community being served” ([1], page 226). Professional skills such as these are in high demand in the sensitive field of palliative care because it involves the treatment of severely ill and dying patients, a situation that often gives rise to difficult conversations. Therefore, palliative-medicine learning contents should be designed to teach the required knowledge, skills and attitude [2] and use different teaching formats. Such formats can include e-learning, accessible via a platform, and designed to teach the necessary knowledge and skills by means of various didactic methods. Former evaluations found that e-learning, in addition to learning gains in knowledge and skills, can also produce changes in attitude [3]. Students value training in palliative care skills, and they especially value the personal meetings with severely ill and dying patients and their relatives [4, 5]. A systematic evaluation of the effectiveness of blended learning formats indicated that such meetings can improve the medical students’ clinical skills and their capacity for reflection [6]. Real -world patient interaction plays a key role in developing those clinical and communication skills [7–9]. The value of involving patients as a positive contribution to basic as well as advanced medical training is already widely recognised. Patient interaction has become an integral part of teaching, learning, and assessment methods [10].

For acquiring complex communication skills like non-verbal communication and empathy, video interaction with real-world patients has proven superior to simulated interaction [11]. Compared to simulated interaction, medical students consider interaction with real-world patients as more instructive and authentic, although simulated interaction can prepare students for real-world situations in that it helps them to develop their communication skills, thereby strengthening their self-confidence [12]. Bokken et al., [12] also provide guidance concerning the decision under which circumstances real-world or simulated interaction should be preferred. Especially in the final phase of medical training, forms of training including both approaches help students rehearse how they should handle difficult situations (e.g., delivery of painful news, dealing with angry patients). In earlier phases of their medical training, their still low levels of clinical skills and knowledge about patient behaviour makes it difficult for the students to develop a professional persona [13]. Furthermore, internal and external aspects of the learning environment are of importance. Where medical training includes more bedside time, students are more willing to learn about end-of-life care, which has a positive impact on the perceived quality of training. In contrast, subliminal negative messages about palliative care from healthcare professionals and patients and their relatives can negatively impact learning [14].

The research quoted in the introduction above show that in later phases of medical training both interaction with real-world patients as well as blended learning can improve the medical students’ clinical and communication skills. At present, there is no published systematic evaluation of changes in knowledge, skills and attitude brought about by palliative care teaching formats that combine real-world patient interaction with blended learning. The objective of this study is a global and outcome-based evaluation of the elective blended-learning course that provides contact with real-world patients. The outcome-based evaluation or outcome assessment aims to objectively evaluate changes in knowledge, skills and attitude among the participants of the elective-course. Furthermore, the study aims to answer the question of whether changes especially in attitude (social skills and self-competence) should be expected after the students have met with severely ill or dying patients.

## Method

### Approach and participants

To meet the many different training needs of new medical students, the Interdisciplinary Centre for Palliative Care at Duesseldorf University Hospital offers students in the clinical training phase a blended e-learning course that includes real-world patient interaction. Each semester, 16 students of human medicine are admitted for participation in this elective course. The course includes preparatory e-learning followed by real-world interaction with patients and concludes with a reflection phase. Throughout the semester-long elective course, instructors are available to answer the students’ questions. In the first 4 weeks of the elective course, the participants engage in e-learning with video-based best practice examples. Course participants can study six e-learning modules that cover the following topics: communication at the end of life; dealing with and assumptions about death and dying, and grief. Some of the best-practice videos feature real patients, while others show actors and actresses simulating patients. Besides professional care providers, some of the conversation partners are also students, apprentices/trainees and pupils from other health-care occupations whom elective-course participants may know and be able to identify. This phase makes it possible for medical students to prepare individually and in their own time for meetings with patients and their relatives. In addition, students are taught factual and application know-how on relevant communication techniques and models. These contents are provided as digital records. Video-based contents and questions designed to encourage reflection on how participants handle their own emotions and attitudes towards dying and death target the affective learning plane, i.e., the students’ personal competence. There are indications that e-learning in palliative medicine exposes attitude-related issues and leads to changes in attitude on ‘palliative thinking’ [15]. In the second phase, participants meet severely ill patients in palliative care and their relatives. The students meet one patient each and can talk to them on one or more occasions, provided that more than one meeting is possible and feasible for both parties. During the semester, students can get support in personalised 1:1 or small-group meetings to discuss the experiences they made in this phase. In the third or follow-up phase the students write a reflection paper that forms the basis for discussion in the subsequent reflection seminar. In this seminar, the students discuss their meetings with patients with their instructors and reflect on their experience.

In the time from April 2018 till March 2020, at the end of each term, i.e., after completing the elective course, all 62 students who had enrolled in the described elective course were invited to participate in this evaluation. Exclusion criteria for participation in the evaluation were completion of the course prior to the designated time and an age < 18 years as well refusal to participate voluntarily. The study included a global teaching evaluation and a specific outcome evaluation. The global teaching evaluation was intended to only evaluate the elective course itself along with its content, structure, and didactic implementation. The specific learning gains were measured using the post-then procedure (i.e., the retrospective comparative self-assessment). All participants gave their informed consent for participation in the study. The study was approved by the ethics committee of Heinrich Heine University Duesseldorf. Data were collected electronically with the Surveymonkey® software.

### Measures

The structured questionnaire for this study included 29 items and was developed specifically in German language. The learning goals of Palliative Medicine, a mandatory subject taught in basic medical training, served as a basis for the development of the questionnaire. They include the ten core competences defined by the European Association of Palliative Care (EAPC) [16] and are based on the German Qualification Framework for Lifelong Learning (Deutscher Qualifikationsrahmen für lebenslanges Lernen, DQR) [17]. According to the DQR, learning goals must reflect both professional skills (i.e., knowledge and skills) as well as personal skills (i.e., social skills and independence/autonomy). Independence/autonomy can be considered as *attitude* and is the ability and willingness to take responsibility for one’s own development and adjustment as a person in terms of social, cultural or professional growth. The DQR describes professional and personal skills as so-called professional competences.

The items were discussed and agreed in the Interdisciplinary Centre for Palliative Medicine’s multi-professional teaching team, who also checked that the contents were clear and easy to understand.

In addition to demographic information (Items I and II) the global evaluation also included 15 items (see Table 1) enquiring about experience from a prior health-care occupation (Item III). Items IV and V deal with the e-learning modules and evaluate their suitability for preparing students for meetings with severely ill patients, and enquire about the clarity of the contents. Three items (VI, VII, XI) judge the structure and didactics of the elective course and how it was carried through. Items VIII and IX evaluate the reflection phase. Another three items (XII, XIII, XIV) measure changes in social and self-competence. Two more items serve the purpose of grading the instructors (Item X) and measuring overall satisfaction (XV) with the elective course. The students rated items on a five-point Likert scale ranging from “Strongly agree” to “Strongly disagree”, where the ratings “Strongly agree” and “Agree” were considered as affirmatives, ie positive responses.

**Table 1:** Global teaching evaluation items of the offered elective course

Question	Subject	Wording
I	Demographics	Gender
II	Demographics	Age
III	Experience from prior health-care occupation	Apart from your medical studies and related internships, have you got any previous experience gained in another a health-care occupation?
IV	E-Learning	The e-learning module is suitable for preparing students for a meeting with a severely ill patient.
V	E-Learning	The contents of the e-learning modules are easy to understand.
VI	Structure	The structure of the elective course dividing it into e-learning, meeting with a patient and subsequent reflection is appropriate.
VII	Implementation	The lecturers/instructors prepared the meetings between the students and the patients well.
VIII	Evaluation of the reflection phase	I found comparing notes during the reflection seminar helpful.
IX	Evaluation of the reflection phase	Writing a reflection paper helps me to cope with my meeting with the patient.
X	Evaluation of lecturers/instructors	The lecturers/instructors appeared enthusiastic.
XI	Didactics	The elective course’s didactic design is appropriate.
XII	Social and self-competence	The elective course has improved my communication skills.
XIII	Social and self-competence	The elective course has changed my attitude towards meeting with dying patients.
XIV	Social and self-competence	I felt overwhelmed/out of my depth during my meeting with a severely ill patient.
XV	Overall satisfaction	Overall, I am satisfied with the elective course.

The outcome-based evaluation of the elective course comprises 14 items (see Table 2). Outcomes are based on the students’ comparative self-assessment of their learning gains. Comparative self-assessment by students is a valid tool for evaluating medical curricula in terms of specific learning goals [18]. Furthermore, CSA is usually less sensitive for construct-irrelevant parameters than student global assessments [19]. The method is used in outcome-based evaluations. In such evaluations, students are asked to retrospectively assess their former and current levels of performance [20].

**Table 2:** Comparative student self-assessment items rated after completing the offered elective course

Question	Wording	Competence
1	I am capable of explaining in detail the basic principles of communication with severely ill patients.	Knowledge
2	I am capable of explaining in detail the NURSE model for dealing with emotions emerging during the meeting.	Knowledge
3	I can actively generate a reassuring atmosphere for my talk with a severely ill patient.	Skills
4	I am confident using non-verbal techniques while talking with a severely ill patient.	Skills
5	I can maintain a neutral attitude in order to professionally handle dying and death in my capacity as a physician.	Attitude
6	I can handle being directly confronted with my own mortality.	Attitude
7	I know how to interpret my dialogue partner’s emotions during a meeting.	Skills
8	I have all the skills and competences I need to be aware of my own feelings and maintain a balance between empathy and professionalism while meeting with a severely ill patient.	Skills
9	I can accept my emotional reaction when sympathising with a patient in an existential crisis.	Attitude
10	I can explain in detail how my own emotions influence my professionalism and work as a physician.	Knowledge
11	I am not afraid of meeting a severely ill patient.	Attitude
12	I know how to choose the right words when meeting with a severely ill patient.	Skills
13	I can handle silence in a meeting with a severely ill patient.	Skills
14	I can treat a dying person with dignity.	Attitude

In retrospective evaluation, the students rated specific learning outcomes on the knowledge plane (Items 1, 2, 10), skills plane (Items 3, 4, 7, 8, 12) as well as social and self-competence plane (Items 5, 6, 9, 11, 13, 14). The 6-point rating scale was the German school grading system (1 = “excellent” till 6 = “unsatisfactory”).

### Data analysis

For the purpose of a global teaching evaluation, a descriptive analysis (frequencies in percent) of the data was performed. The specific learning outcome as measured with comparative self-assessment was determined using the following formula [18]:

CSA gain[%] = ((mvpre-mvpost)/(mvpre-1)) × 100.

mvpre = mean level of self-assessed competence before starting the elective course.

mvpost = mean level of self-assessed competence after completion of the elective course.

## Results

### Descriptive statistics

In the time from April 2018 till March 2020 a total of 62 students participated in the global elective course evaluation and the specific outcome evaluation. None of the study participants had to be excluded from the global or the specific evaluation because of incomplete data. Almost two thirds (69.8%) of the participants were female; most students (58%) were between 20 and 24 years old. Approximately one third (31.1%) of the students had experience gained in a previous health-care occupation. On average, students accessed the e-learning modules 14.2 times (range: 1–30 times) and spent a mean time of 6.5 h (range: 2–20 h) with the offered contents.

### Global evaluation

The e-learning part received very positive ratings: 98.4% of the students found it useful in preparing them for their patient meetings, and all students found the contents easy to understand. All students found the elective course’s structure and didactics meaningful and felt that their teachers had adequately prepared them for their meetings with patients (96.8%) The students stated that both their communication skills (93.6%) and their attitude towards dealing with dying patients (90.3%) had improved or changed. Overall, 98.4% of the participants were very satisfied with the elective course.

### Outcome evaluation

According to the outcome-based evaluation the highest learning gains were achieved on the knowledge plane, i.e. the explanation of the NURSE model (Naming, Understanding, Respecting, Supporting, Exploring) [21], skills plane, i.e. the active generation of a reassuring meeting atmosphere, and attitude plane, i.e. the ability to meet a severely ill patient without fear (for details see Table 3). The lowest learning gain was observed in the application of non-verbal communication techniques during the meetings. Across all items, the highest learning gains were observed in knowledge, followed by skills, and, finally, social and self-competence (for details see Table 4).

**Table 3:** Students’ self-reported learning gains, percentage

Item	Mean value pre	Mean value post	CSA gain
1	3.7	2.1	59.3%
2	3.5	2.0	60.0%
3	2.9	1.7	63.2%
4	2.7	1.8	54.3%
5	3.0	2.0	50%
6	2.9	2.3	31.6%
7	2.3	1.8	38.5%
8	2.7	1.9	47.1%
9	2.8	2.0	44.4%
10	2.8	2.0	44.4%
11	3.2	1.8	63.3%
12	3.8	2.5	46.4%
13	2.8	1.8	55.6%
14	1.8	1.5	37.5%

*N* = 62

**Table 4:** Percentage of learning gains in professional competence according to DQR

	Professional skills		Personal skills
	Knowledge	Skills	Social and self-competence
Questions	1, 2, 10	3, 4, 7, 8, 12	5, 6, 9, 11, 13, 14
Learning gains	54.6%	49.9%	47.1%

*N* = 62

## Discussion

The presented study is an outcome assessment that performs a general teaching evaluation on the elective course and measures a specific learning outcome. The offered elective course helps students to broaden their knowledge and skills and supports them in achieving social and self-competence development goals. Outcomes showed that the combination of real-world patient meetings and digital learning formats can prompt students to change their attitudes.

Overall, the concept of blended learning in the teaching of palliative medicine is rated as positive by the students. The global evaluation shows that students confront, and reflect upon, the topics of death and dying. This outcome matches findings by [6] and supports existing data demonstrating that e-learning is an effective means for acquiring professional competence [22–24]. The existing data also show that e-learning is an accepted method among medical students [22]. The evaluated elective course with real-word patient interaction supports medical students not only in acquiring knowledge and communication skills, it also encourages the development of a personal mindset and emotional engagement with topics like death and dying. The offered course succeeded in reaching the participating students on the attitude plane and resulted in learning gains that prepared them for their future profession and the involved interaction with severely ill patients. This finding supports the research results achieved by [14]. The authors were able to show that a higher proportion of personal patient interaction prompts students to systematically learn about patient care at the end of life. In addition, real-world patient interaction is conducive to the development of interpersonal skills [7–9]. In our study, learning gains were achieved in the domain communication skills, specifically in generating a reassuring atmosphere for the meetings with patients. In our opinion, this requires suitable non-verbal communication as well as self-confidence. Hearing directly from the patients how important reassurance is for them in situations like these gives a boost to learning gains. Previous studies showed real-world patient interaction is more effective when students need to learn complex communication skills (e.g. body language) [11]. Furthermore, outcomes also pinpointed those areas of interaction with severely ill or dying patients where young students need special support. This study, for example, found relatively small learning gains in the appropriate interpretation of the patients’ emotions (required for feeling empathy). According to the students, however, the level of empathy at this point was already high to begin with, so limits to the extent of learning gains could be expected. Based on the outcome of just one single item, the highest learning gain in this evaluation study was achieved in the domain attitude. After completing the elective course, students were able to meet severely ill patients with much less fear, which was also the reason why they enrolled in the elective course in the first place. In their reflection papers and in the reflection seminars, many students stated that they enrolled in the elective course out of concern about how to address severely ill patients in order to deliver painful news or discuss their situation, and expressed their wish to acquire the necessary skills. During the meetings, the students learned that the patients frequently wanted physicians to be honest, so all involved could have a frank discussion of any issues at hand. This experience can help students build confidence and mitigate fears caused by the challenges that such meetings pose.

However, the highest learning gains across all items were achieved knowledge plane. Previous studies evaluating the use of e-learning with Best-Practice examples in the teaching of palliative medicine found similar outcomes [3]. In this evaluation study, however, the learning gains on the competence plane were even higher. Even though real-world patient interaction appears to be a useful tool for encouraging changes in attitude, the question of which methods are particularly suitable for building social and self-competence remains open due to lack of empirical evidence.

### Limitations

One limitation of this study is its small sample size and the purely descriptive analysis of data. Despite validation of the CSA gain method, the students’ comparative self-assessment does not allow a wholly objective evaluation either. Furthermore, a comparison between different sub-groups (e.g., with or without experience from a prior health-care occupation) would have been interesting for result interpretation. Due to the small size of the study population we decided to perform a general analysis and did not define any sub-groups. Given the small number of students participating in this evaluation, a sub-group comparison would not have been representative. For data privacy reasons, we could not perform sub-group analysis on the e-learning modules, either. It is not permitted to simultaneously record virtual e-learning attendance and subsequent evaluation. This means we can see how much time students spend in e-learning but we cannot match evaluation responses to individual persons. All considered, a longer observation period and a significance test as well as validation of the questionnaires used would have helped to make the results representative. In addition, combining a quantitative design with the collection of additional qualitative data could contribute to improved outcome significance. For this purpose, the reflection papers written by the students could be included the evaluation.

## Conclusions

Our evaluation of the offered elective course showed that students achieve learning gains in knowledge, skills and attitude. Furthermore, outcomes also highlighted those areas of interaction with severely ill or dying patients where young students need special support. In the long term, higher-level medical communications skills can potentially improve the doctor-patient relationship and, as a result, improve a patient’s quality of life at the end of his/her life.

## Data Availability

The datasets used and/or analysed during the current study are available from the corresponding author on request.

## Abbreviations

E-learning: Electronic learning technology
CSA: Comparative self-assessment
CSA gain: Comparative self-assessment gain
MS: Manuela Schallenburger
TT: Tabea Thyson
AS: Alexandra Scherg
JS: Jacqueline Schwartz
AL: Anne Leister
MN: Martin Neukirchen
